# RETRACTED: Rehman et al. Piperine Regulates Nrf-2/Keap-1 Signalling and Exhibits Anticancer Effect in Experimental Colon Carcinogenesis in Wistar Rats. *Biology* 2020, *9*, 302

**DOI:** 10.3390/biology14050464

**Published:** 2025-04-25

**Authors:** Muneeb U. Rehman, Summya Rashid, Azher Arafah, Wajhul Qamar, Rana M. Alsaffar, Ajaz Ahmad, Nada M. Almatroudi, Saeed M. A. Alqahtani, Shahzada Mudasir Rashid, Sheikh Bilal Ahmad

**Affiliations:** 1 Department of Clinical Pharmacy, College of Pharmacy, King Saud University, P.O. Box 2457, Riyadh 11451, Saudi Arabia; aazher@ksu.edu.sa (A.A.); aajaz@ksu.edu.sa (A.A.); 2 Division of Veterinary Biochemistry, Faculty of Veterinary Science and Animal Husbandry, SKUAST-Kashmir, Alustang, Shuhama 190006, J&K, India; drsmrashid786@gmail.com; 3 Department of Pharmacology & Toxicology, College of Pharmacy Girls Section, Prince Sattam Bin Abdulaziz University, P.O. Box 173, Al-Kharj 11942, Saudi Arabia or s.abdulrashid@psau.edu.sa (S.R.); r.alsaffar@psau.edu.sa (R.M.A.); 4 Department of Pharmacology & Toxicology, College of Pharmacy, King Saud University, P.O. Box 2457, Riyadh 11451, Saudi Arabia; wqidris@ksu.edu.sa (W.Q.); 439106181@student.ksu.edu.sa (S.M.A.A.); 5 Department of Clinical Pharmacy, College of Pharmacy Girls Campus, King Saud University, P.O. Box 2457, Riyadh 11451, Saudi Arabia; 439204163@student.ksu.edu.sa

The journal retracts the article, “Piperine Regulates Nrf-2/Keap-1 Signalling and Exhibits Anticancer Effect in Experimental Colon Carcinogenesis in Wistar Rats” [1] cited above.

Following publication, concerns were brought to the attention of the publisher regarding duplication with images from a different publication [2] and an overlap of sub-images within one of the figures in this publication [1].

Adhering to our standard procedure, an investigation was conducted by the Editorial Office and Editorial Board that confirmed the presence of duplicated panels between Figure 3A,B in this publication [1] and Figures 2A and 4A in the published article [2] with at least one shared author. In addition, a partial overlap between sub-images within Figure 7 was also confirmed.

While the authors fully cooperated with the Editorial Office during the investigation, they were unable to satisfactorily explain the duplication and overlap of figures, nor provide raw data for the Editorial Board’s evaluation. As a result, the Editorial Board has lost confidence in the reliability of the findings and decided to retract this paper [1] as per MDPI’s retraction policy (https://www.mdpi.com/ethics#_bookmark30).

This retraction was approved by the Editor-in-Chief of the journal *Biology*.

The authors wish to communicate to the readers that they were unable to reproduce the results of the study and therefore requested its retraction.
